# Detection of nucleic acids and other low abundance components in native bone and osteosarcoma extracellular matrix by isotope enrichment and DNP-enhanced NMR†

**DOI:** 10.1039/c9ra03198g

**Published:** 2019-08-27

**Authors:** Ieva Goldberga, Rui Li, Wing Ying Chow, David G. Reid, Ulyana Bashtanova, Rakesh Rajan, Anna Puszkarska, Hartmut Oschkinat, Melinda J. Duer

**Affiliations:** Department of Chemistry, University of Cambridge Lensfield Road Cambridge CB2 1EW UK mjd13@cam.ac.uk; Leibniz-Forschungsinstitut für Molekulare Pharmakologie (FMP) Campus Buch, Robert-Roessle Str. 10 Berlin 13125 Germany oschkinat@fmp-berlin.de

## Abstract

Sensitivity enhancement by isotope enrichment and DNP NMR enables detection of minor but biologically relevant species in native intact bone, including nucleic acids, choline from phospholipid headgroups, and histidinyl and hydroxylysyl groups. Labelled matrix from the aggressive osteosarcoma K7M2 cell line confirms the assignments of nucleic acid signals arising from purine, pyrimidine, ribose, and deoxyribose species. Detection of these species is an important and necessary step in elucidating the atomic level structural basis of their functions in intact tissue.

## Abstract 2

Towards elucidating their biological roles in intact tissue, DNP NMR reveals nucleic acids, and other important low abundance biomolecules in a complex biomaterial, bone, and in cancer extracellular matrix.

## Graphical abstract

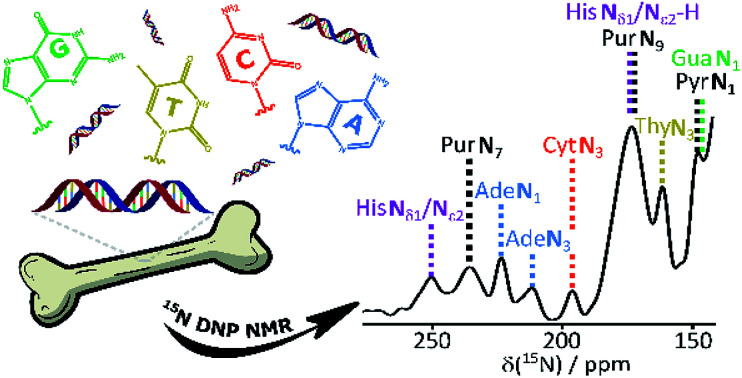

The extracellular matrix (ECM) is a complex, heterogeneous, and active, environment, playing structural roles, but also strongly influencing cell behaviour in both homeostasis and pathology. The difficulty of understanding the composition and regulation of the ECM parallels the situation within cells, but with the added complication that many molecular components are insoluble. Solid-state NMR (ssNMR) can contribute to a better understanding of the ECM through direct detection of sparse species, made possible by a strategy of isotopic enrichment of native biomaterials^1^ combined with rapidly evolving dynamic nuclear polarization (DNP) enhancement techniques.^2^ Briefly these are based on the transfer of the much greater electron spin polarization, excited by a microwave laser at the electron resonance frequency of an organic free radical dopant, to the (NMR active) nuclei of the molecules of interest, at low temperatures to maximize polarization. The conversion of electron to nuclear polarization results in NMR sensitivity enhancements of up to several orders of magnitude. Recently we have reported direct observation of important low abundance collagen post-translational modifications (PTMs) in intact native, and model, ECM.^3^ While we focussed on species derived from one specific amino acid (Lys) component of the ECM, it is also of great potential importance to investigate whether other sparse ECM species become observable with DNP enhancement. Non-protein minor species, besides low abundance amino acids, are of particular interest since they represent a realm in which NMR can provide atomic length scale molecular structural information complementing other current biophysical techniques. Incorporating non-protein molecules into the overall structural biology picture will broaden conceptions of which ECM components to investigate in further detail towards an integrated model of the biological and material properties of tissues.

Here, we report DNP-enhanced NMR on mouse bone^1^ enriched in a wide range of ^13^C, ^15^N-amino acids. Where necessary to distinguish between low abundance protein, and non-protein, signals, we confirmed amino acid type assignments by comparison with ECM produced by cells cultured on media supplemented with one or a few specific amino acids, using methods already described.^4,5^ Our previous analysis of bone focussed on the relatively abundant species observable with conventional ssNMR. The enhancement provided by DNP now reveals non-protein biomolecules biosynthesized from isotopically enriched amino acids and/or sugars, and their incorporation into bone. The preparation of stable isotope enriched biomaterials, and our DNP NMR methodology, is described in detail in the ESI,† Materials and methods sections.

We begin our investigation on bone with a suite of ssNMR characterizations under DNP signal enhancement conditions. A full 1D ^15^N DNP NMR spectrum is shown in ESI Fig. S1.† Apart from the predictable major signals (from backbone and sidechain amides, and abundant Lys and Arg) the sensitivity enhancement of enrichment-DNP reveals a number of more minor resonances (Fig. 1A) from lower abundance species; the shifts of many of those at high spectral frequency coincide closely with the common (deoxy)nucleotide base ring nitrogens (Biological Magnetic Resonance Database, BMRB, as summarized in ESI Schemes S1† (DNA) and S2 (RNA)). Signals from the nitrogen atoms of protonated His imidazolium (*ca.* 180 ppm) and unprotonated imidazole (*ca.* 250 ppm) ring forms are also observed.^6,7^ The His assignments are reinforced by ^15^N data from model osteoblast (bone precursor) ECM selectively enriched in (U-^13^C_6_, ^15^N_3_)-His (His*) (Fig. 1B; see ESI† for preparation and comparison of ^15^N–^13^C correlation spectra, Fig. S2†). The low frequency region of the bone DARR-assisted ^15^N–^13^C correlation spectrum (Fig. 1C) displays a clear cross peak (65 ppm ^13^C, 52 ppm ^15^N) which we ascribe to the (CH_3_)_3_**N̲**^+^–**C̲**H_2_–CH_2_–O– correlation in the head groups of phosphatidylcholine (PC) lipids (^13^C assignments based on Kroon *et al.*;^8^ see ESI Fig. S3† for overlays with model compound spectra). The PC ethanolamine substructure is biosynthesized from a single serine unit so, in our bone material, all atoms will be concurrently labelled. Finally Fig. 1C also shows the distinctive Hyl N_ζ_–C_δ_ fingerprint correlations; the close correspondence with (U-^13^C_6_, ^15^N_2_)-Lys enriched ECM from vascular smooth muscle cells^3^ is shown in ESI Fig. S4,† proving that our labelling – DNP NMR combination readily detects the Hyl PTM even in this complex organic–inorganic composite native biomaterial, as well as in simpler native (skin) and model ECMs.^3^ Chemical shifts are summarized in Table 1.

**Fig. 1:**
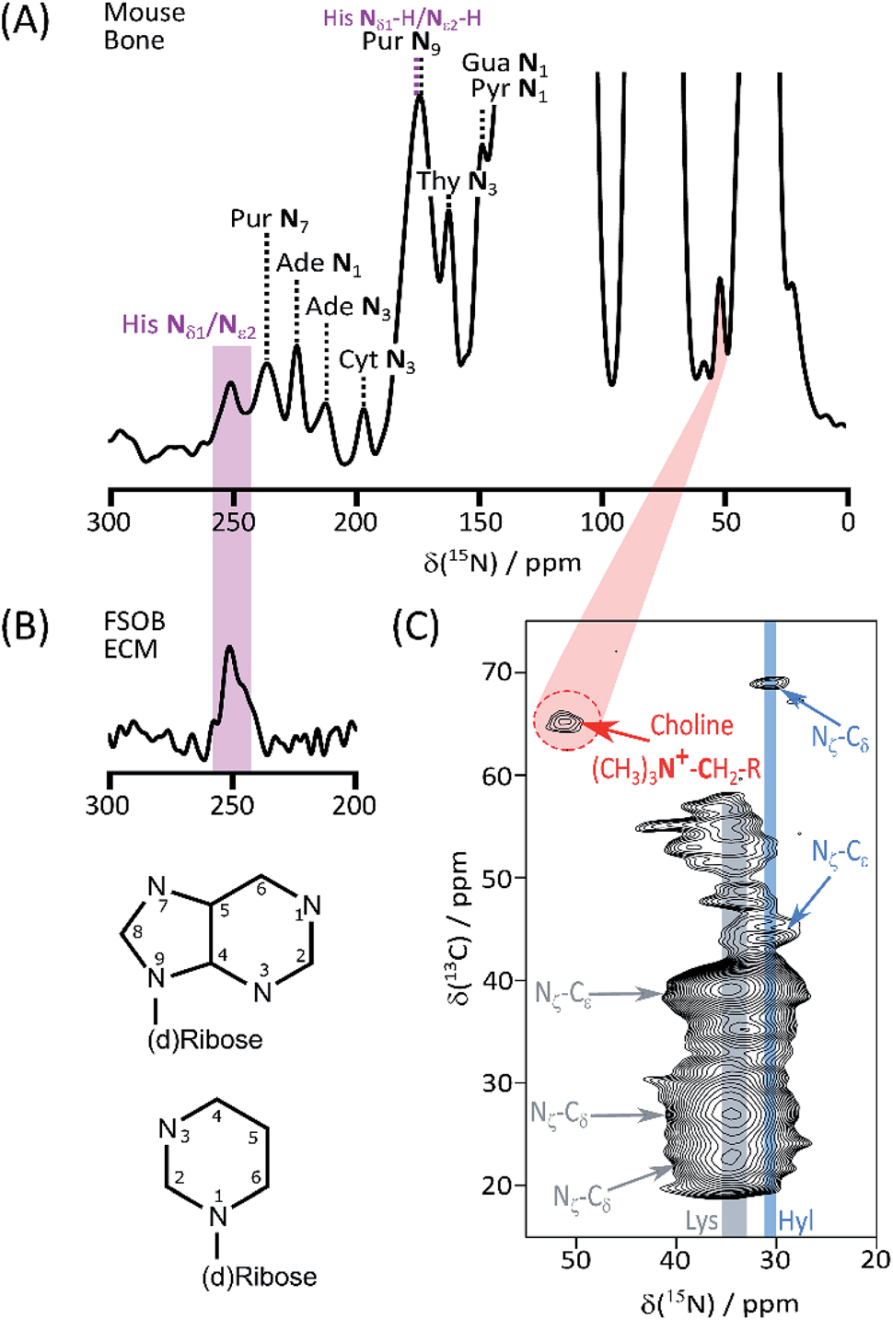
(A) High frequency region of the ^15^N DNP-NMR spectrum showing assignments of resolved base signals of nucleic acids, and ring His signals; (B) ^15^N spectrum of His* FSOb ECM highlighting correspondence with the signal from the imidazole form of the His ring; (C) DARR assisted ^15^N–^13^C correlation spectrum of labelled bone, transmitting magnetization along the Hyl (and Lys) sidechain to emphasize correlations between Hyl (as well as the intense Lys) N_ζ_ and C_δ_. Also highlighted is the signal from the (CH_3_)_3_**N̲**^+^–**C̲**H_2_–CH_2_–O– correlation from phosphatidylcholine headgroups. Atom numbering of the nucleic acid purine (top) and pyrimidine skeletons is also shown.

**Table 1:** Chemical shifts of resolved signals from low abundance species detected in ^13^C, ^15^N enriched bone by DNP NMR

**Amino acids/PTMs**						
Hyl	C_δ_	C_ε_	N_ζ_			
	69	44	31			
His	C_γ_	C_δ2_	C_ε1_	N_δ1_^a^	N_ε2_^a^	N_δ1/ε2_^b^
	129	117	135	180	177	250

**(Deoxy)nucleotide bases**						
		N_1_	N_3_	N_7_	N_9_	
Ade		224	212	235^c^	174^c^	
Gua		148		235^c^	174^c^	
Cyt		148	197		197	
Thy/Ura		162	148			

**Lipids**						
PC	–**N̲**(CH_3_)_3_^+^	–**C̲**H_2_–N^+^				
	52	65				

a Protonated imidazolium form.

b Unprotonated imidazole form.

c Single unresolved signal.

Detection of His, lipid components, and Hyl, in native biomaterials is not unexpected. In contrast nucleic acids in bone have been little discussed, although this tissue is a favourable source of forensic genetic material.^9^ Given this underrepresentation of bone in nucleic acid literature, we turned to ECM from the aggressively metastasizing K7M2 osteosarcoma cell line^10^ to substantiate these signals. A ^13^C DNP NMR spectrum of K7M2 ECM supplemented with (U-^13^C_2_, ^15^N)-Gly (Gly*) and (U-^13^C_6_)-glucose (Glc*) is shown in Fig. 2A; it shows a number of clear signals with shifts consistent with the common (deoxy)nucleotide bases^11^ (ESI Schemes S1 and S2†). Abundant nucleic acid in the K7M2 ECM is confirmed by conventional *in situ* propidium iodide staining and confocal fluorescence microscopy (ESI Fig. S5†). The biosynthetic routes to the bases (ESI Scheme S3†) dictates that all the base carbon atoms will be at least partially ^13^C enriched by Gly* and Glc*, although only the purine C_4_–C_5_–N_7_ substructure (from intact Gly*), and to some extent pyrimidine C_4_–C_5_–C_6_ from Glc* *via* Ser,^12^ will necessarily be concurrently labelled. (Deoxy)riboses are biosynthesized from Glc* and so all (deoxy)sugar rings will be concurrently ^13^C enriched. Fig. 2B details results of a through-bond (J-transmitted) INADEQUATE,^13^ which clearly shows the predicted purine C_4_–C_5_ and pyrimidine C_4_–C_5_ cross peaks. DARR^14^ (Fig. 2C) confirms these and indicates pyrimidine C_6_ signals in Cyt and Thy, and Ura from RNA. The presence of both DNA dRib, and RNA Rib, is confirmed by the low frequency part of the INADEQUATE spectrum (Fig. 2D). ^15^N DNP NMR shows a clear signal from purine N_7_'s, corresponding closely to that of the putative purine N_7_'s in bone (Fig. 2E). Also a ^13^C{^15^N} TEDOR^15^ (Fig. 2F) mapping short carbon-nitrogen distances, shows the expected purine N_7_–C_5_ and weaker N_7_–C_4_ correlations, but also purine N_7_–C_8_ cross peaks (the latter atom acquired from Glc* *via N*-formyl tetrahydrofolate). All DNA and RNA shifts measured in K7M2 ECM are summarized in Table 2.

**Fig. 2:**
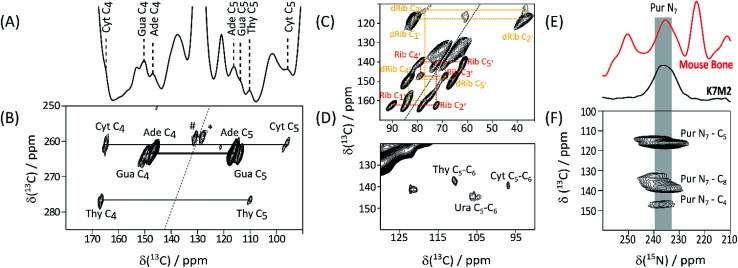
^13^C DNP NMR spectra of Gly*, Glc* supplemented K7M2 ECM. (A) ^13^C CP-MAS; nucleotide base signals assigned by 2D experiments and database shift values (ESI Schemes S1 and S2†) are labelled; (B) high frequency region of INADEQUATE J-transmitted SQ-DQ correlation spectrum (265 ppm ^13^C DQ sweep width to avoid aliasing; ^#,^ * – correlations between lipid olefinic carbons); (C) low frequency region of the same spectrum tracing out correlations within ribose (Rib), and deoxyribose (dRib), rings; (D) high frequency region of the ^13^C–^13^C DARR correlation spectrum base; (E) ^15^N DNP NMR of Gly*Glc* K7M2 ECM compared with that of labelled bone, supporting the assignment of the 235 ppm signal as purine N_7_; (F) ^13^C{^15^N} TEDOR spectrum showing the expected purine N_7_–C_5_ and N_7_–C_4_, as well as the putative purine ring N_7_–C_8_, through-space correlations (^15^N sweep width 438 ppm to cover full ^15^N range).

Chemical shifts of resolved (deoxy)nucleotide signals in the K7M2 rat osteosarcoma cell line

**Table:** 

	C2	C4	C5	C6	C8	N7
Ade	153^a^	147^b^^,^^c^	116^b^^,^^c^	153^a^	139^b^^,^^e^	236^b^^,^^e^
Gua	153^a^	150^b^^,^^c^	114^b^^,^^c^	167^a^	136^b^^,^^e^	239^b^^,^^e^
Cyt	153^a^	165^c^	96^c^	140^d^		
Thy	153^a^	167^c^	110^c^	137^d^		
Ura			105^d^	145^d^		

a Signals observed in 1D spectrum although no SQ-DQ or DARR correlations observable; assignments are inferred from average shifts reported in BMRB.

b Assignments to Ade and Gua are based on rank order of BMRB average shifts, and may be reversed.

c From J-transmitted INADEQUATE SQ-DQ correlation.

d From ^13^C–^13^C DARR correlation.

e From ^15^N–^13^C TEDOR.

**Table:** 

	C1′	C2′	C3′	C4′	C5′
dRib	82	37	78	84	65
Rib	90	72	69	79	61

There are large number of catalogued nucleic acid shifts in the BMRB but as yet no robust equivalent of the chemical shift index^16^ of protein secondary structural elements, especially with respect to ^15^N. Nucleotide base ^13^C and ^15^N shifts are particularly sensitive to secondary structural features^17–20^ but linewidths and overlap in our ^13^C and ^15^N spectra currently preclude structural conclusions about K7M2 ECM. In principle though this work suggests that the potential of ^15^N shifts as indices of nucleic acid structure^20,21^ in whole tissues can be realized through several conformational “reporter” nitrogen atoms in the well resolved high frequency NMR window above 150 ppm. Nucleic acid in bone may be predominantly intracellular, unlike that of K7M2 where a higher extracellular proportion is expected, but it is interesting that these distinctive ^15^N signals can be observed in both systems. We envisage that detection by DNP-NMR in native tissue is an essential step in fulfilling this potential to characterize nucleic acid structure in the intact tissue and ECM environment.

NMR detection of nucleic acids in bone raises fresh questions about their biological role, and avenues to new insights. NMR detection is an essential first step in elucidating these roles at the molecular and atomic level in native materials. Extracellular roles *e.g.* signalling, of nucleic acids are likely to operate in K7M2 ECM, and potentially also in bone and bone diseases *e.g.* osteoporosis.^22^ Bone undergoes constant remodelling so a high degree of transcriptional, translational, and apoptotic activities are expected,^23^ leading to significant amounts of nucleic acid being produced in bone cells and released in the ECM. Once in the extracellular environment, some nucleic acids may have a different function in addition to their intracellular roles. For instance, nucleic acids have pronounced affinity for apatite mineral,^24^ and may participate in biomineralization,^25^ as calcium sequestering and mineral templating scaffolds, a role that is already proposed for structurally related polynucleotides like poly-ADP-ribose.^4^

Phospholipids^26^ are abundant in mineralized bone.^27^ They may represent the presence of matrix vesicles – extracellular structures *ca.* 100 nm in size, “pinched off” from the outer membranes of osteoblasts – which act as a nidus for initial calcium phosphate precipitation in normal^28^ and pathological^29^ biomineralization. The molecular mechanisms underlying the initial bioprecipitation events and processes remain to be elucidated, in particular the role of matrix vesicle phospholipids. Direct observation of the PC headgroup by NMR in healthy bone is a significant step in atomic level understanding of the relevant intermolecular and interionic interactions. In addition to matrix vesicles, of 40–100 nm sized exosomes are the basis of a pathway for cancer metastasis^30^ and therapy resistance^31^ through delivery of proteins, lipids, RNA and DNA to remote cells.^32^ The cargo carried by these vesicles may play unexpected roles if released directly in the extracellular space and allowed to interact with other components of the ECM.^33^ DNP-enhanced NMR is potential non-targeted method of better understanding the role played by these extracellular vesicles.

Hyl and its subsequent modifications are essential to the structure, properties, and functions, of collagen.^34^ The type I collagen heterotrimer contains 12 His residues. Both of those in each α1 (I) chain are two residues from those Lys residues which are sequence specifically δ-hydroxylated to Hyl, in a strongly conserved Lys-Gly-His-Arg tetrad.^35^ These His residues have been proposed to play a part in targeting lysyl hydroxylases and thereby the formation of the specific Lys and Hyl crosslinks^36^ essential to maintaining collagen and tissue structure. Some of the same His residues are not only hydroxylated to Hyl, but of these some are subsequently also enzymatically glycosylated to (Glc)GalHyl, an essential event in directing the as-yet poorly understood process of collagen fibril assembly. It has recently been proposed that these self same residues are also preferentially glycated in non-enzymatic, usually harmful, reactions with metabolic sugars.^37^ Finally there is evidence for a trivalent crosslink, histidinohydroxylysinonorleucine, between these same Lys/Hyl and His residues.^38^ Thus His and Lys/Hyl residues co-exist in crosslinking and glycosylation/glycation “hotspots” which may be key to better understanding the spatial relationships underlying orderly, and pathological, collagen assembly and structure. Detecting His, Lys and Hyl simultaneously, as enabled by isotopic enrichment and DNP-enhanced NMR, in native and model ECM, opens fertile new possibilities for studying these processes.

This communication demonstrates again^3^ the ability of ssNMR, combined with signal enhancement by DNP and targeted isotope labelling, to detect low abundance components in intact tissue that may have surprising biological roles, thus complementing other biophysical and molecular biology techniques in exploring diverse areas of matrix biology.

## Conflicts of interest

There are no conflicts of interest. Animal procedures were unregulated in terms of the U. K. Animals (Scientific Procedures) Act 1986, but nevertheless were reviewed for compliance with institutional ethical guidelines by the Animal Welfare Ethical Review Board, University of Cambridge.

## Supplementary Material

RA-009-C9RA03198G-s001

## References

[cit1] Wong V. W. C., Reid D. G., Chow W. Y., Rajan R., Green M., Brooks R. A., Duer M. J. (2015). J. Biomol. NMR.

[cit2] Thankamony A. S. L., Wittmann J. J., Kaushik M., Corzilius B. (2017). Prog. Nucl. Magn. Reson. Spectrosc..

[cit3] Chow W. Y., Li R., Goldberga I., Reid D. G., Rajan R., Clark J., Oschkinat H., Duer M. J., Hayward R., Shanahan C. M. (2018). Chem. Commun..

[cit4] Chow W. Y., Rajan R., Muller K. H., Reid D. G., Skepper J. N., Wong W. C., Brooks R. A., Green M., Bihan D., Farndale R. W., Slatter D. A., Shanahan C. M., Duer M. J. (2014). Science.

[cit5] Li R., Rajan R., Wong W. C. V., Reid D. G., Duer M. J., Somovilla V. J., Martinez-Saez N., Bernardes G. J. L., Hayward R., Shanahan C. M. (2017). Chem. Commun..

[cit6] Li S. H., Hong M. (2011). J. Am. Chem. Soc..

[cit7] Hu F., Schmidt-Rohr K., Hong M. (2012). J. Am. Chem. Soc..

[cit8] Kroon P. A., Quinn D. M., Cordes E. H. (1982). Biochemistry.

[cit9] Kitayama T., Ogawa Y., Fujii K., Nakahara H., Mizuno N., Sekiguchi K., Kasai K., Yurino N., Yokoi T., Fukuma Y., Yamamoto K., Oki T., Asamura H., Fukushima H. (2010). Leg. Med..

[cit10] Khanna C., Khan J., Nguyen P., Prehn J., Caylor J., Yeung C., Trepel J., Meltzer P., Helman L. (2001). Cancer Res..

[cit11] Sergeyev I. V., Day L. A., Goldbourt A., McDermott A. E. (2011). J. Am. Chem. Soc..

[cit12] Nelson D. L. and Cox H. M., Lehninger: Biochemistry, W. H. Freeman & Co., New York, 6th edn, 2013

[cit13] Lesage A., Auger C., Caldarelli S., Emsley L. (1997). J. Am. Chem. Soc..

[cit14] Takegoshi K., Nakamura S., Terao T. (2001). Chem. Phys. Lett..

[cit15] Jaroniec C. P., Filip C., Griffin R. G. (2002). J. Am. Chem. Soc..

[cit16] Wishart D. S. (2011). Prog. Nucl. Magn. Reson. Spectrosc..

[cit17] Borer P. N., LaPlante S. R., Zanatta N., Levy G. C. (1988). Nucleic Acids Res..

[cit18] LaPlante S. R., Boudreau E. A., Zanatta N., Levy G. C., Borer P. N., Ashcroft J., Cowburn D. (1988). Biochemistry.

[cit19] Gaffney B. L., Goswami B., Jones R. A. (1993). J. Am. Chem. Soc..

[cit20] Xu X. P., Au-Yeung S. C. F. (2000). J. Phys. Chem.
B.

[cit21] Gao X., Jones R. A. (1987). J. Am. Chem. Soc..

[cit22] Yavropoulou M. P., Yovos J. G. (2018). J. Musculoskeletal Neuronal Interact..

[cit23] Jilka R. L., Noble B., Weinstein R. S. (2013). Bone.

[cit24] Loreille O. M., Diegoli T. M., Irwin J. A., Coble M. D., Parsons T. J. (2007). Forensic Sci. Int.: Genet..

[cit25] Coscas R., Bensussan M., Jacob M. P., Louedec L., Massy Z., Sadoine J., Daudon M., Chaussain C., Bazin D., Michel J. B. (2017). Atherosclerosis.

[cit26] Boskey A. L., Timchak D. M. (1983). Metab. Bone Dis. Relat. Res..

[cit27] Haraszti R. A., Didiot M. C., Sapp E., Leszyk J., Shaffer S. A., Rockwell H. E., Gao F., Narain N. R., DiFiglia M., Kiebish M. A., Aronin N., Khvorova A. (2016). J. Extracell. Vesicles.

[cit28] Golub E. E. (2009). Biochim. Biophys. Acta.

[cit29] Hsu H. H. T., Camacho N., Aono H. (1999). Atherosclerosis.

[cit30] Williams C., Rodriguez-Barrueco R., Silva J. M., Zhang W. J., Hearn S., Elemento O., Paknejad N., Manova-Todorova K., Welte K., Bromberg J., Peinado H., Lyden D. (2014). Cell Res..

[cit31] Li I., Nabet B. Y. (2019). Mol. Cancer.

[cit32] Lagerweij T., Perez-Lanzon M., Baglio S. R. (2018). J. Vis. Exp..

[cit33] Min L., Shen J., Tu C. Q., Hornicek F., Duan Z. F. (2016). Cancer Metastasis Rev..

[cit34] Myllyharju J. (2005). Top. Curr. Chem..

[cit35] Rodriguez-Pascual F., Slatter D. A. (2016). Sci. Rep..

[cit36] Helseth D. L., Lechner J. H., Veis A. (1979). Biopolymers.

[cit37] Hudson D. M., Archer M., King K. B., Eyre D. R. (2018). J. Biol. Chem..

[cit38] Mechanic G. L., Katz E. P., Henmi M., Noyes C., Yamauchi M. (1987). Biochemistry.

