# Supplementary material for: Detection of nucleic acids and other low abundance components in native bone and osteosarcoma extracellular matrix by isotope enrichment and DNP-enhanced NMR
Source: RSC Adv. 2019 Aug 27;9(46):26686–90. doi: 10.1039/c9ra03198g (PMC9070537; doi:10.1039/c9ra03198g)
Supplement: RA-009-C9RA03198G-s001 [file RA-009-C9RA03198G-s001.pdf]

Electronic Supplementary Material (ESI) for RSC Advances.
This journal is © The Royal Society of Chemistry 2019

Electronic Supplementary Information (ESI)

Detection of nucleic acids and other low abundance components in native bone and osteosarcoma extracellular matrix by isotope enrichment and DNP-enhanced NMR

Ieva Goldberga,[a] Rui Li,[a] Wing Ying Chow,[b] David G. Reid,[a] Ulyana Bashtanova,[a] Rakesh Rajan,[a] Anna M. Puszkarska,[a] Hartmut Oschkinat,*[b] Melinda J. Duer*[a]

a. IG, RL, DGR, UB, RR, AMP, MJD* - Department of Chemistry, University of Cambridge, Lensfield Road, Cambridge CB2 1EW, UK

b. WYC, HO - Leibniz-Forschungsinstitut für Molekulare Pharmakologie (FMP), Campus Buch, Robert-Roessle Str. 10, Berlin 13125, Germany

* Author for correspondence: mjd13@cam.ac.uk, Telephone: +44(0)1223-736394, Fax: +44(0)1223-336362

Materials and methods **p. 2**

Scheme S1 **p. 5**

Scheme S2 **p. 7**

Scheme S3 **p. 8**

Figure S1 **p. 10**

Figure S2 **p. 11**

Figure S3 **p. 12**

Figure S4 **p. 13**

Figure S5 **p. 14**

References **p. 15**

Materials and Methods

Labelling of mouse tissue[1]

Labelled diet (2000 g modified Classic A03 Geldiet (SAFE, Augy, France) comprising 50 g ^{13}C,^{15}N-labelled Celtone powder (Cambridge Isotope Laboratories, Andover, MA, U. S. A.), 67 g fish hydrolysate, 410 g corn starch, 72.9% water, and 2.1% preservatives and texture additives) to female C57Bl/6 mice during pregnancy and lactation until weaning of pups. At this point all mice were humanely euthanized using a Schedule 1 method and tissues harvested and stored at -80° C until acquisition of NMR data. The *in vivo* phase of the project complied with the institutional Ethical Review Process, and the U. K. Animals (Scientific Procedures) Act 1986.

Isolation of Fetal Sheep Osteoblasts[2]

Fetal sheep femoral osteoblasts were isolated as follows: After thorough washing (1% trigene, Medichem International), muscle and non-osseous tissue was stripped away and bone sectioned into small longitudinal pieces which were washed with 70% ethanol then with Minimum Essential Medium (MEM; Invitrogen), transferred to Dulbecco's Modified Eagle Medium (DMEM; Invitrogen) containing bacterial collagenase A (0.5 mg/mL) and dispase II (3 mg/mL) (Roche Diagnostics), and incubated at 37°C with shaking for 3 hours to release osteoblasts into the medium. The bone sections were rinsed in DMEM with 20% fetal calf serum (FCS; Invitrogen) to halt enzyme activity, rinse medium and cell suspension pooled and passed through a 40 µm mesh filter (Appleton Woods), centrifuged at 1000 g for 5 min at room temperature. The resulting pellet was resuspended in DMEM complete medium and transferred to two T-175 cm^3 culture flasks (Nunc) and placed in a 37°C CO_2 incubator. When nearly confluent, cells were detached with 10 ml of 0.25% trypsin containing 1 mM EDTA (SigmaAldrich) and incubated for 5 min at room temperature. The flasks were tapped at the end of incubation period to completely dislodge the cells from the flask. Trypsin was neutralized by adding 15 mL of DMEM complete media to the culture flask. The cell suspension was centrifuged in a 50 mL tube (Greiner) at 1200 rpm for 5 min and resuspended in 10 mL of DMEM. The cells were transferred into T-175 cm^3 culture flasks and were expanded to passage 3 for subsequent experiments.

Basal Medium Eagle (BME) complete medium was prepared by adding 10% FCS, 30µg/mL L-ascorbic acid 2-phosphate (Sigma), 10mL/L L-glutamine-penicillin-streptomycin (200 mM L-glutamine, 10,000 units/ml penicillin, and 10 mg/ml streptomycin in 0.9% sodium chloride; Sigma). DMEM complete medium was prepared by adding 10% FCS, 30µg/mL L-ascorbic acid 2-phosphate, and 10mL/L L-glutamine-penicillin-streptomycin. All supplements were filter sterilized (0.22 µm filter, Appleton Woods) before addition.

Culturing osteoblasts with labelled compounds

Osteoblasts were cultured to confluence in T-175 flasks containing 25 mL BME complete medium. Labelled (U-$^{13}C_6$,$^{15}N_3$) histidine.HCl (Cambridge Isotope Laboratories) was added to a final concentration of 42 mg/L after filter sterilization (0.22 µm filter). The culture was incubated at 37°C in a humidified atmosphere of 95% air and 5% CO_2. The medium with isotope labelled supplement was renewed every 2 days until the cells and matrix began to detach at nine days, at which point enough ECM had formed for SSNMR. The medium was removed and the cells were washed with 20 mL 1 x phosphate buffered saline (1X PBS). The flask was placed in a freezer at -80ºC for 24 hours and the cells were lysed by thawing at room temperature for 30 minutes. The resultant debris was removed by repeated washes with PBS and decellularized ECM dislodged by gently swirling the flask with 20 mL PBS. The matrix collected in PBS was transferred to a fresh 50 mL tube and centrifuged at 1200 rpm

for 5 min at room temperature. The supernatant was poured off and the ECM dehydrated in an oven at 37ºC overnight. The samples were stored at -20°C until NMR analysis.

Culturing K7M2 mouse osteosarcoma

K7M2 cells (ATCC) were cultured in T-175 flasks containing 25 mL complete Dulbecco's Modified Eagle Medium (DMEM; 4.5g/L glucose; Invitrogen). Complete DMEM was composed of 10% foetal calf serum (Pan Biotech), 30 µg/mL L-ascorbic acid 2-phosphate (Sigma) and 10 mL/L L-glutamine-penicillin/streptomycin (200 mM L-glutamine, 10,000 units/ml penicillin, and 10 mg/ml streptomycin in 0.9% sodium chloride; Sigma). All supplements were filter sterilized (0.22 µm filter, Appleton Woods) before addition. On the next day of seeding cells, medium was changed, and labelled (U-^{13}C, ^{15}N) glycine (Cambridge Isotope Laboratories) and (U-^{13}C) glucose (Cambridge Isotope Laboratories) were added after filter sterilization (0.22 µm) to a final concentration of 90 mg/L and 9 g/L respectively in the complete medium. The culture was incubated at 37°C in a humidified atmosphere of 95% air and 5% CO_2. The culture medium with isotope labelled supplements was renewed every 2 days until cells were confluent and flask surface was fully covered by its matrix (take around 10 days). Afterwards, the medium was removed and the cells were washed twice with 10 mL phosphate buffered saline (1X PBS, Invitrogen). The flask was placed in a freezer at -80°C for 24 hours and the cells were lysed by thawing the flask at room temperature for 30 minutes. The debris produced by cell lysis was removed by repeated washes with water, and the rest of matrix was lyophilized overnight. The samples were stored at -20°C until NMR analysis.

Rotor packing for DNP-NMR experiments

Specific conditions for each sample:

1. **Mouse pup forelimb bone**, enriched in ^{13}C,^{15}N-labelled amino acids.
Sample mass: 13.9 mg (intact pieces)
0.174 mg of solid AMUPol[3] was added to 12 µL of water (12 µL D2O, 3 µL H2O) to yield a 19.9 mM AMUPol solution. 10 µL of this solution was added to the solid sample in the rotor.
DNP enhancement, e = 22 ± 1.

2. Gly*,Glc*-K7M2 cell line ECM
Sample mass: 12 mg (freeze dried)
0.49 mg of solid bcTol-M[4] was added to 20 µL of water (14 µL D2O, 6 µL H2O) to yield a 39.5 mM bcTol-M solution. 5 µL of this solution was added to the solid sample in the rotor. e = 26 ± 9

3. Lys*-VSMC ECM
Sample mass: 10.0 mg (freeze dried)
0.445 mg of solid AMUPol was added to 30 µL of water (22.5 µL D2O, 7.5 µL H2O) to yield a 20.4 mM AMUPol solution. 12.5 µL of this solution was added to the solid sample in the rotor. e = 57 ± 4

4. His*-FSOB ECM
Sample mass: 29.3 mg (wet)
0.428 mg of solid AMUPol was added to 24 µL of water (24 µL D2O, 8 µL H2O) to yield an 18.4 mM AMUPol solution. 10 µL of this solution was added to the solid sample in the rotor. e = 16 ± 6

For all NMR experiments, 3.2 mm outer diameter zirconia rotors were filled and closed with a Vespel cap, then spun with the cap upwards, then downwards, on a desktop mini-centrifuge to distribute the

radical through the sample. The packed rotors were equilibrated with the radical solution overnight at 4°C before the start of DNP NMR experiments.

DNP NMR spectroscopy

DNP NMR was performed on a 9.4T Bruker Avance III wide-bore 400 MHz spectrometer equipped for DNP NMR with a gyrotron operating at 9.7 T and a 3.2 mm (^{1}H-^{13}C-^{15}N) triple resonance low-temperature magic angle spinning (LTMAS) probe. The MAS rate was 8889 Hz unless otherwise noted, and sample temperature 110 ± 5 K at a LTMAS cooling cabinet gas flow of 2000 litres per hour. Experimental conditions were optimized for solid state DNP NMR of biomolecular systems.[4]

NMR parameters were: ^{1}H 90° pulse length 2.2-2.5 µs, ^{13}C 90° pulse length 4.1-4.75 µs, 80 kHz ^{1}H decoupling, DNP enhancement achieved with 30 mA microwave irradiation. Widely used ^{1}H-^{13}C cross polarisation (CP) techniques based on dipolar coupling and specifically detecting immobile and solid species generated initial ^{13}C magnetization using a 1500 µs ^{1}H-^{13}C ramped CP[5] contact time.

Dipolar-assisted rotary resonance (DARR) ^{13}C-^{13}C 2D homonuclear correlation: This experiment uses the transfer of magnetization between ^{13}C atoms via intervening protons, to correlate carbon atoms which are close to each other (but not necessarily bonded) in space. It is analogous to the well known NOESY experiment in liquid state NMR. Spectra were obtained with a DARR mixing sequence[6] with mixing times of 5 and 20 ms, which probe shorter, and longer, ^{13}C-^{13}C distances respectively, with experiments typically signal averaged for 7-15 hours.

Dipole-dipole transmitted single quantum-double quantum (SQ-DQ) ^{13}C-^{13}C homonuclear correlation: This experiment relies on the direct transfer of magnetization between nearby ^{13}C atoms without magnetization proceeding via intervening protons. Effectively it correlates only 13C atoms which are bonded to each other because the ^{13}C-^{13}C nuclear magnetic dipole interaction is comparatively weak and falls off rapidly with internuclear distance. The data are usually presented as 2D contour plots in which the single quantum x-axis ("F2 axis" in NMR convention) represents the conventional 1D NMR spectral chemical shifts, and the double quantum y-axis ("F1 axis") which represents the sum of the shifts of the pairs of bonded atoms, i.e. if two atoms with chemical shifts d1 and d2 are bonded, this will show as a pair of cross peaks at chemical shift coordinates of (δ_1, $\delta_1+\delta_2$) and (δ_2, $\delta_1+\delta_2$). Experiments were initiated by CP followed by a SPC5[7] mixing sequence. The MAS rate was 8300 Hz to avoid overlap of signals with spinning sidebands. DQ coherence was excited by applying the SPC5 sequence for 1.2 ms (10 times). Experiments were typically signal averaged for 6 hours.

Heteronuclear ^{15}N-^{13}C correlation experiments were carried out by two methods, double CP and transferred echo double resonance (TEDOR).[8] The usual 2D contour plot presentation of both types of experiment is analogous to well known solution state heteronuclear correlation experiments such as HSQC and HMBC. The two axes represent 1D ^{13}C and ^{15}N chemical shifts, and cross peaks correspond to pairs of ^{13}C-^{15}N atoms which are close to each other in space. Representative 90° pulse lengths were: ^{1}H 2.4 µs, ^{13}C 4.33 µs, ^{15}N 4.27 µs.

Double CP followed by DARR mixing: Double CP transfers magnetization between nearby ^{15}N and ^{13}C nuclei. Because the ^{15}N – ^{13}C internuclear magnetic dipole interaction is weak, in its simplest form this experiment essentially only correlates directly bonded nitrogen and carbon atoms. The addition of a DARR step which transfers magnetization from the directly N-bonded to more distant ^{13}C atoms effectively probes ^{15}N relationships with more carbon atoms. As in the ^{13}C-^{13}C DARR correlation experiment already described, increasingly larger nitrogen-carbon distances can be probed by

increasing the DARR mixing time. Magnetization was first excited by ^{1}H-^{15}N CP with a 450 μs contact pulse length, with a slight ramp shape on the ^{15}N channel, followed by indirect ^{15}N evolution, during which ^{13}C magnetisation was refocussed by a 90°-180°-90° sequence and ^{1}H was decoupled at 80 kHz. Magnetization was then transferred by ^{15}N-^{13}C SPECIFIC-CP[9,10] with a 4.5 ms contact pulse length, with a square shape on ^{15}N and a tangential shape on ^{13}C. At this point there was no indirect evolution on ^{13}C, instead, a DARR sequence (mixing time of 10 ms) was immediately applied on the ^{13}C channel, followed by ^{13}C acquisition with 80 kHz ^{1}H decoupling.

TEDOR: This experiment is analogous to the double CP experiment just described except that the method of generating internuclear magnetization transfer is different. In a normal magic angle spinning experiment the ^{13}C-^{15}N internuclear magnetic dipole interaction, which is distance sensitive, is significantly reduced (to achieve sharp spectral lines); in the TEDOR experiment this dipole interaction is restored ("recoupled") by a series of 180° pulses on one of the nuclei (^{15}N here) which are applied synchronously with the MAS period i.e. "rotor synchronized". By increasing the recoupling period, more distant carbon-nitrogen distances can be probed. As TEDOR depends on direct ^{13}C-^{15}N dipolar interactions and is not transmitted via intervening protons, it is useful in characterization of species such as nucleic acid bases without abundant attached protons. Magnetization was first excited by ^{1}H-^{13}C CP with an 800 μs contact pulse length, with a 100%-70% ramp shape on the ^{1}H channel. A rotational echo double resonance (REDOR) sequence followed, with a rotor-synchronized spin echo on ^{13}C and two dephasing π pulses on ^{15}N for every rotor cycle. Then a z-filter was used to remove anti-phase elements prior to INEPT-like transfer of the magnetisation to ^{15}N and indirect evolution. Thereafter, rotor synchronicity was re-established as the magnetisation was returned to ^{13}C. A second REDOR sequence followed. A z-filter is applied again prior to ^{13}C acquisition with 90 kHz ^{1}H decoupling.

Refocussed INADEQUATE ^{13}C-^{13}C 2D SQ-DQ homonuclear correlation:[11] This experiment differs from the preceding ones in that magnetization is transferred between ^{13}C atoms via the through-bond ("J", or "scalar"), homonuclear coupling familiar in solution state NMR, and not via the usual (in solid state NMR) through-space internuclear dipole-dipole interaction. The 2D presentation of the data is exactly equivalent to that of the dipole-dipole transmitted SQ-DQ experiment already described. Magnetization was first excited by ^{1}H-^{13}C CP with an 800 μs contact pulse length, with a 100%-70% ramp shape on the ^{1}H channel. The refocussed INADEQUATE sequence (τ - 180° - τ - 90° - t1 - 90° - τ - 180° - τ) was then applied on the ^{13}C channel, with the τ delay set to 3.5 ms. The INADEQUATE sequence and ^{13}C acquisition were carried out with 90 kHz ^{1}H decoupling.

Scheme S1 Averaged ^{13}C and ^{15}N chemical shifts of the common nucleotides in **DNA** (statistics based on the BMRB http://www.bmrb.wisc.edu/ref_info/stats.php?set=filt&restype=dna&output=html). Black numbers – atom numbering convention; red numbers - mean chemical shifts, from the BMRB.

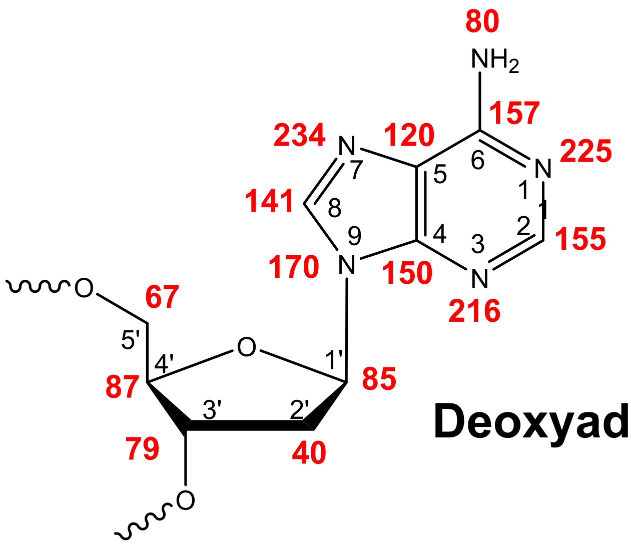

Deoxyadenosine

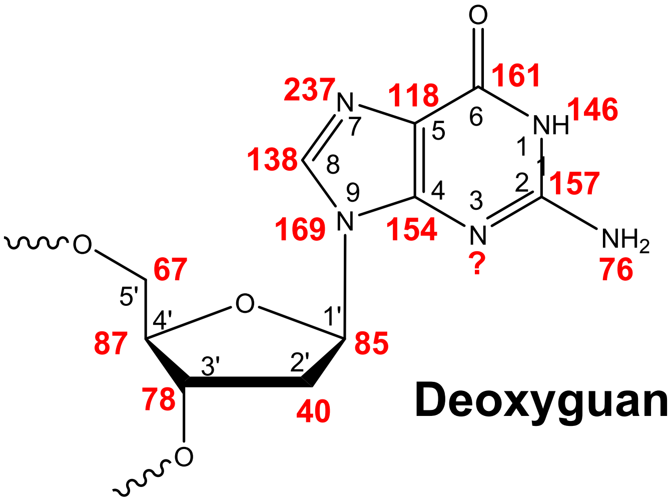

Deoxyguanosine

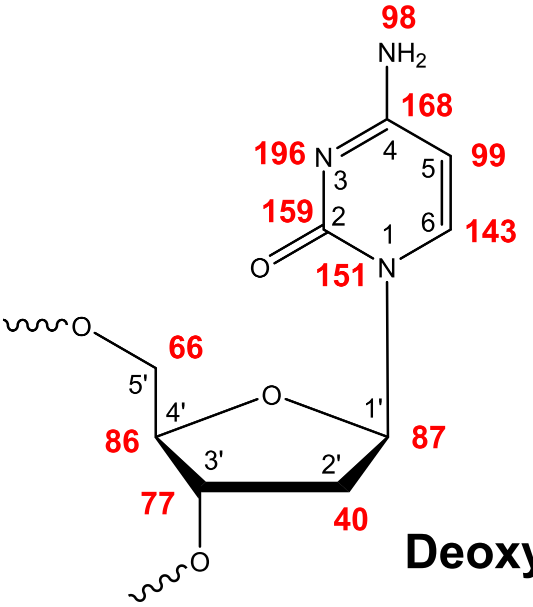

Deoxycytidine

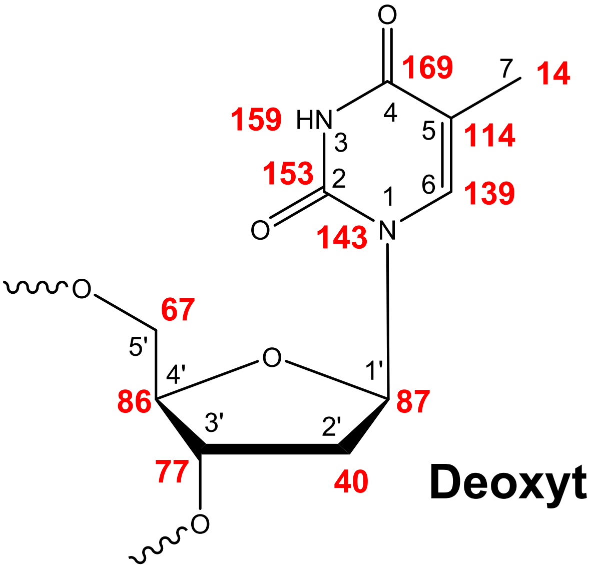

Deoxythymidine

Scheme S2 Averaged ^{13}C and ^{15}N chemical shifts of the common nucleotides in **RNA** (statistics based on the BMRB http://www.bmrb.wisc.edu/ref_info/stats.php?set=filt&restype=dna&output=html). Black numbers – atom numbering convention; red numbers - mean chemical shifts, from the BMRB.

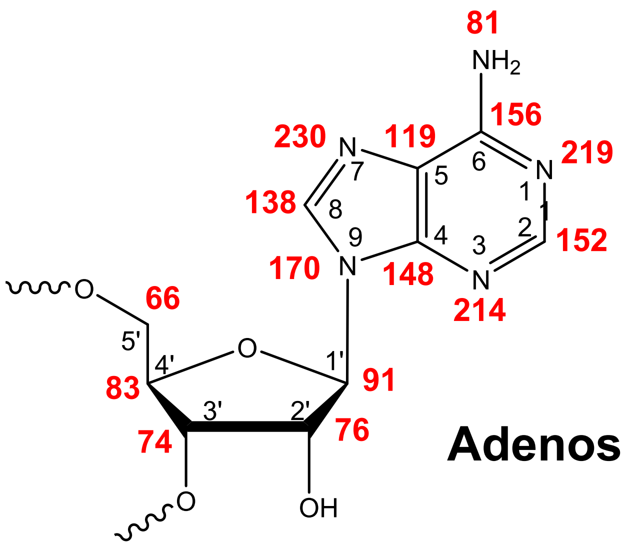

Adenosine

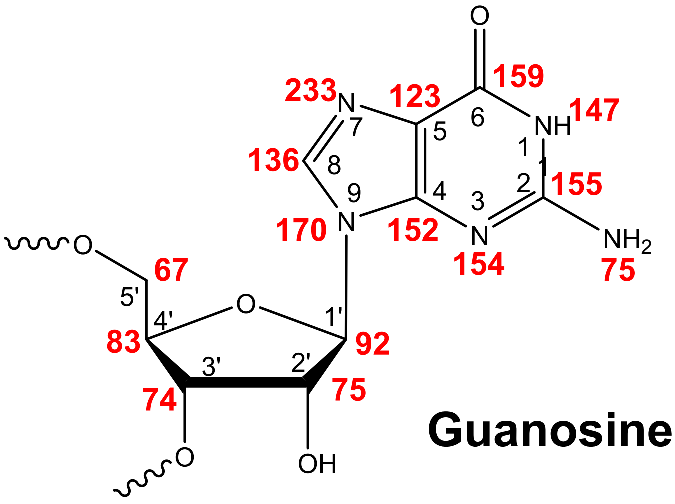

Guanosine

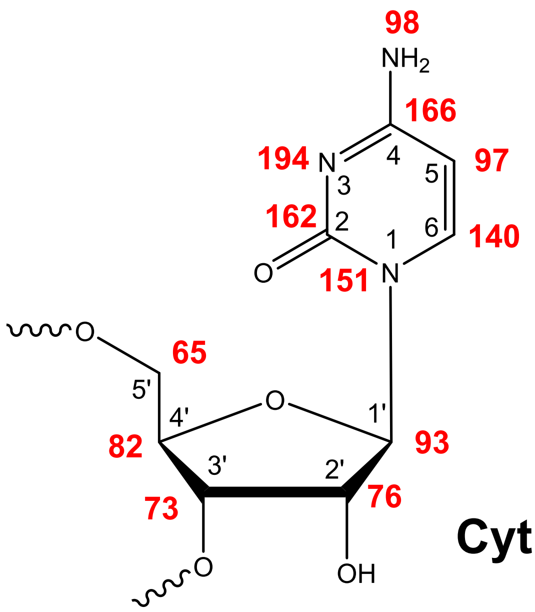

Cytidine

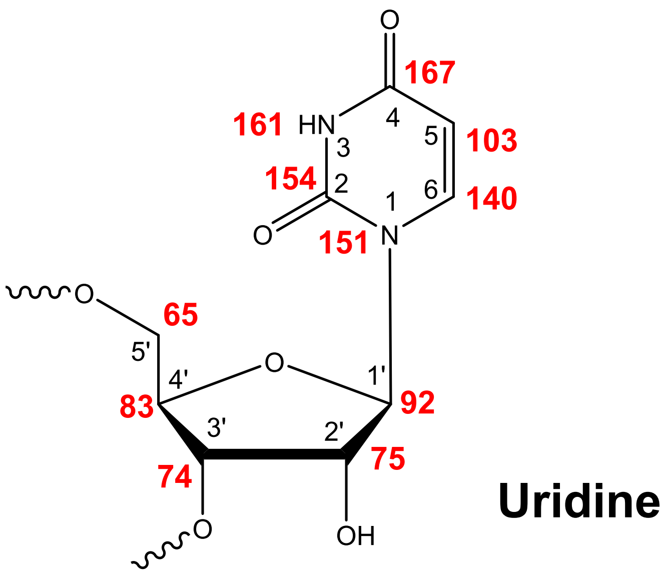

Uridine

Scheme S3 Biosynthetic origin of nucleic acid skeleton purine (left) and pyrimidine (right) atoms. THF – Tetrahydrofolate.

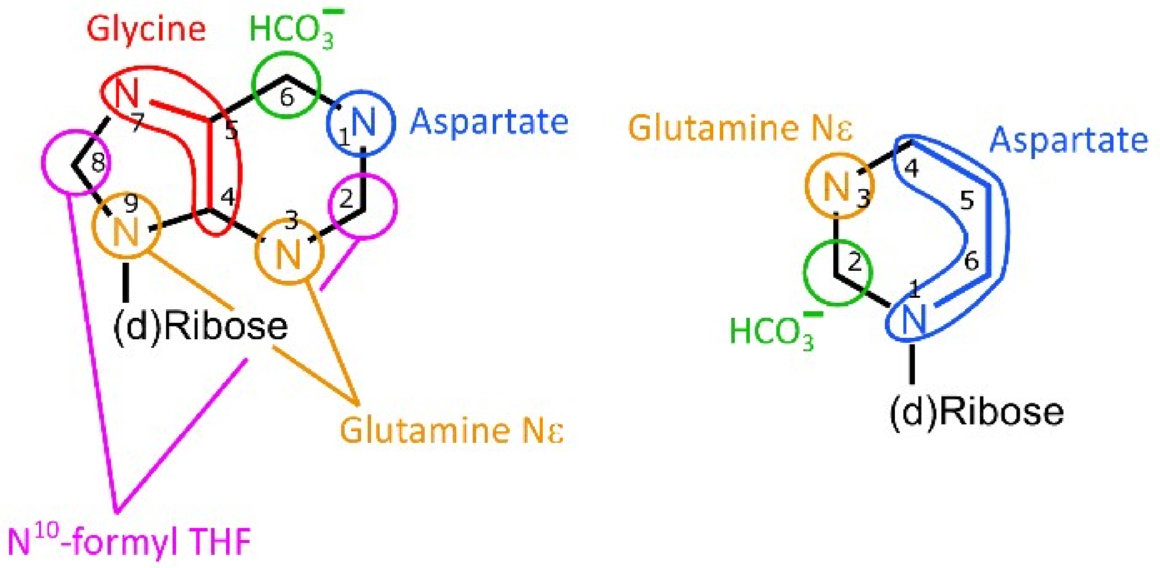

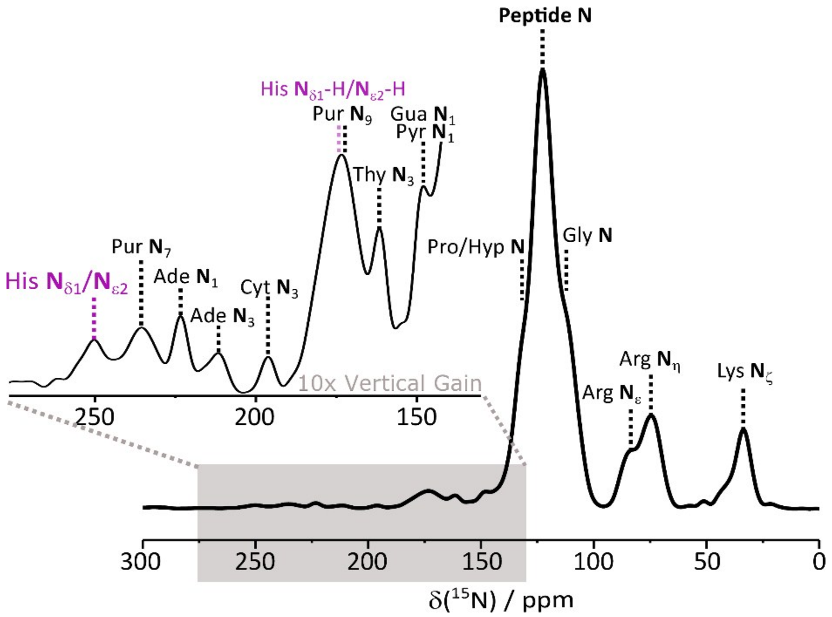

Figure S1 ^{15}N DNP NMR of ^{13}C ^{15}N-labelled amino acid-enriched mouse forelimb bone. Assignments of the major signals by chemical shift is straightforward; minor signal assignments in the expanded inset correspond to those shown in Fig. 1A.

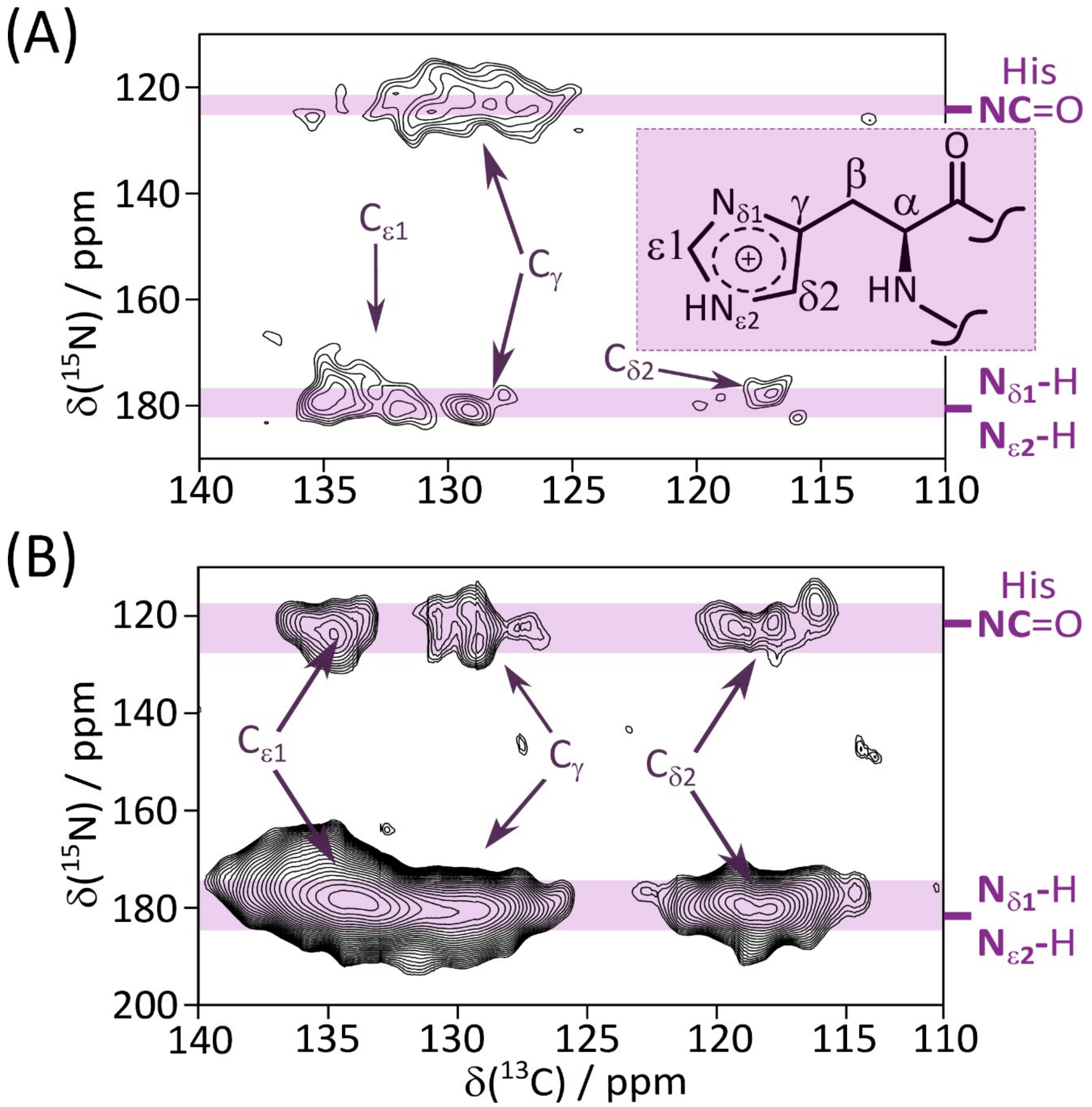

Fig. S2 ^{13}C – ^{15}N correlations of His (protonated, i.e. imidazolium, form) in labelled bone (A), and (B) in His*-labelled osteoblast matrix. As mentioned in the Materials and Methods section, the F2 (i.e. x) and F1 (i.e. Y) axes of the contour plots correspond to ^{13}C and ^{15}N chemical shifts respectively. For instance the contour set labelled "$C_{\varepsilon 2}$" in (B) is evidence of the proximity of the ε2 imidazolium carbon (135 ppm) to its almost degenerate neighbour $N_{\delta 1}$ and $N_{\varepsilon 2}$ atoms (177-180 ppm), as well as to the more distant His backbone nitrogen (ca. 122 ppm).

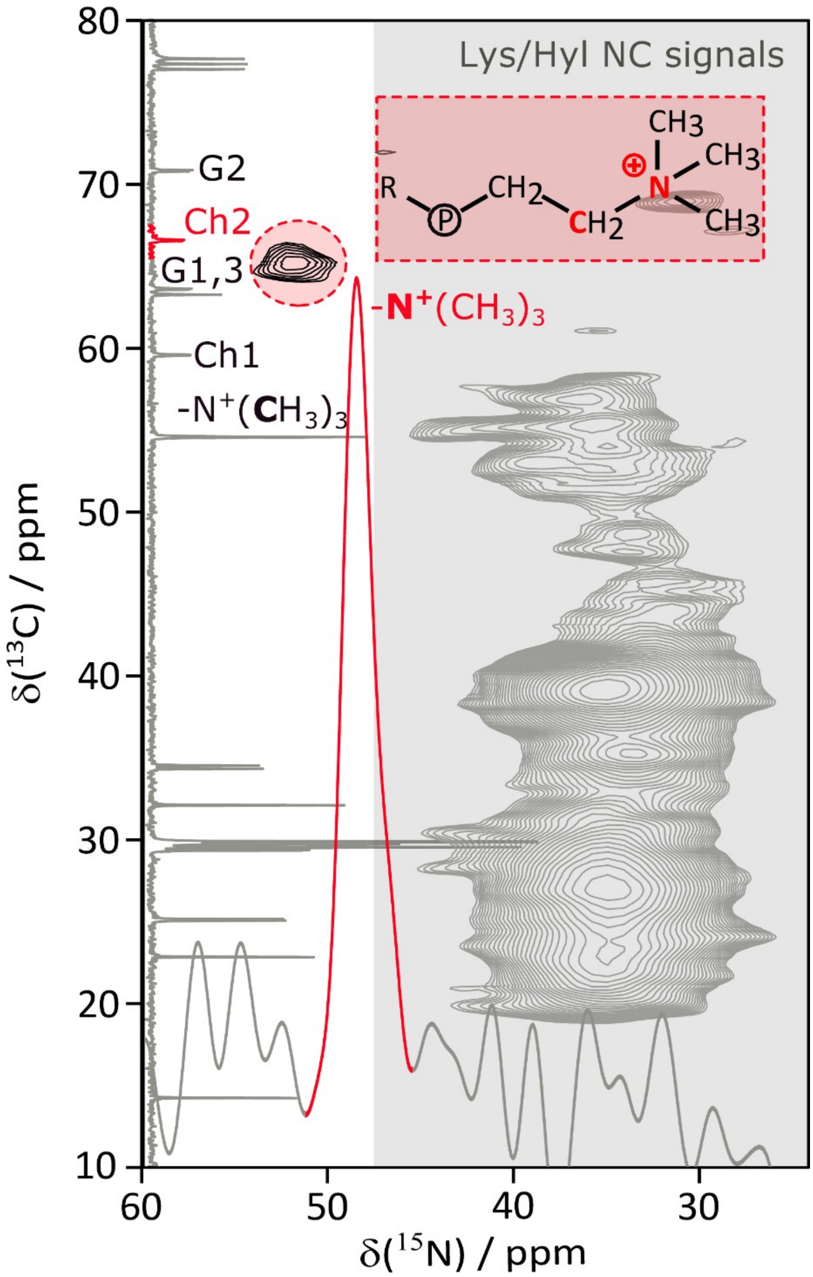

Figure S3 Overlay of lower frequency ^{15}N – ^{13}C correlations in labelled bone, with solution state ($CDCl_3$) ^{13}C spectrum of the model phospholipid dimyristoylphosphatidyl choline (DMPC; Sigma, horizontal overplotted 1D spectrum), and the solid state room temperature ^{15}N spectrum of DMPC (vertical overplotted 1D spectrum). The putative choline (Ch) headgroup -**C2** – **N**$^+$(CH_3)$_3$ correlation is circled. There is a close although not exact correspondence between the ^{13}C (signal labelled Ch2) and ^{15}N shifts of the model compounds, and those of bone; we ascribe these small discrepancies to factors such as: liquid state NMR (^{13}C of DMPC), and variations in DMPC packing and headgroup orientation (^{13}C and ^{15}N). R – Diacylglycerol; G – Glycerol.

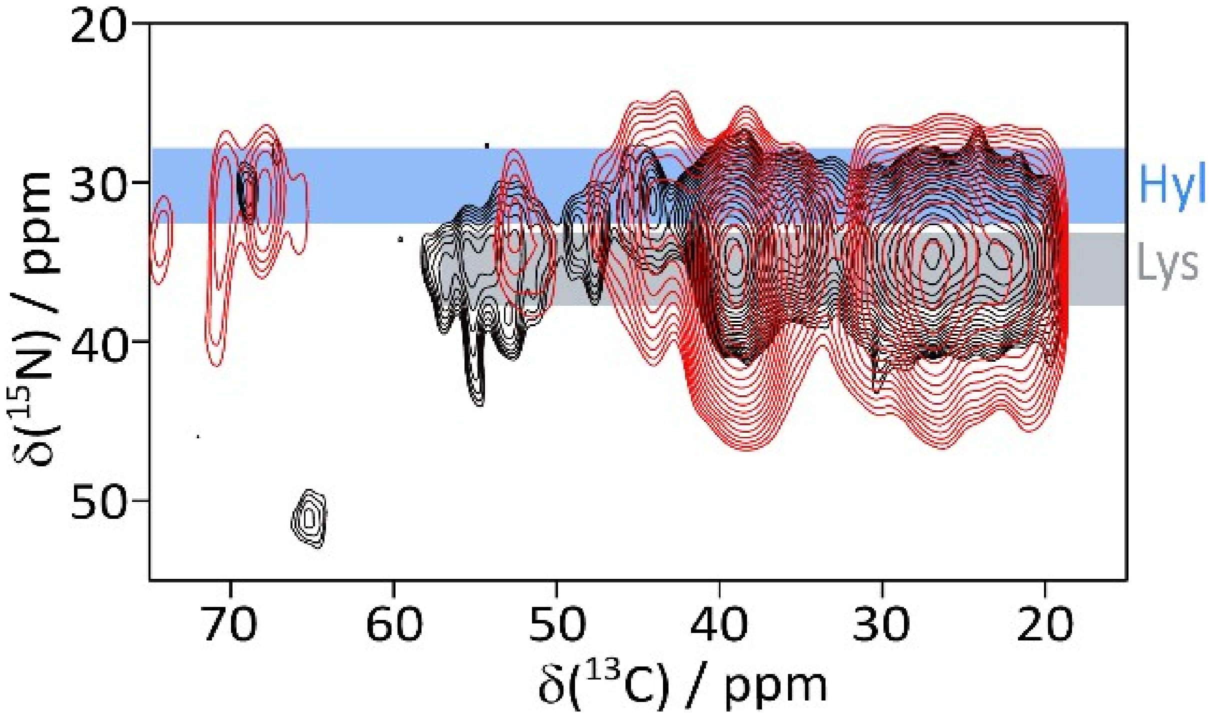

Figure S4 Overlay of DARR-assisted ^{15}N – ^{13}C double CP correlations in labelled bone (black contours), and Lys*-VSMC ECM (red contours), rotated to be consistent with our recent presentation of the latter,[12] illustrating the correspondence of the "fingerprint" Hyl - C_{δ}-N_{ζ} (ca. 70 ppm ^{13}C, ca. 30 ppm ^{15}N) and other Hyl correlations between the two materials.

Fig. S5 Confirmation of abundant nucleic acid in K7M2 ECM Partially decellularized K7M2 ECM was stained with propidium iodide, a fluorescent nucleic-acid intercalating dye. Defrosted matrix was incubated in 50μg/mL propidium iodide in PBS at room temperature for 15 min and washed twice. Images (Cambridge Advanced Imaging Centre; Leica TCS SP8 confocal microscope) were taken in frame sequential mode, as follows: blue autofluorescence excitation 405/ emission 415 -500 nm (blue channel) and propidium iodide excitation 561/ emission 590-690nm (red channel). Weak matrix autofluorescence was detected across the whole visual range of excitation/emission (only blue and red channels are shown) and fully overlapped (see the resultant purple coloration of overlaid images (Fig. S5 P and L)). Such a broad autofluorescence range is characteristic of cellular lipid degradation products e.g. lipofuscin. Propidium iodide revealed DNA inside the numerous nuclei in the K7M2 matrix (Fig. S5 B and F); thread-like extranuclear staining not overlapping with autofluorescence (Fig. S5 D and H) can be attributed to RNA, and/or DNA, leached from damaged nuclei.

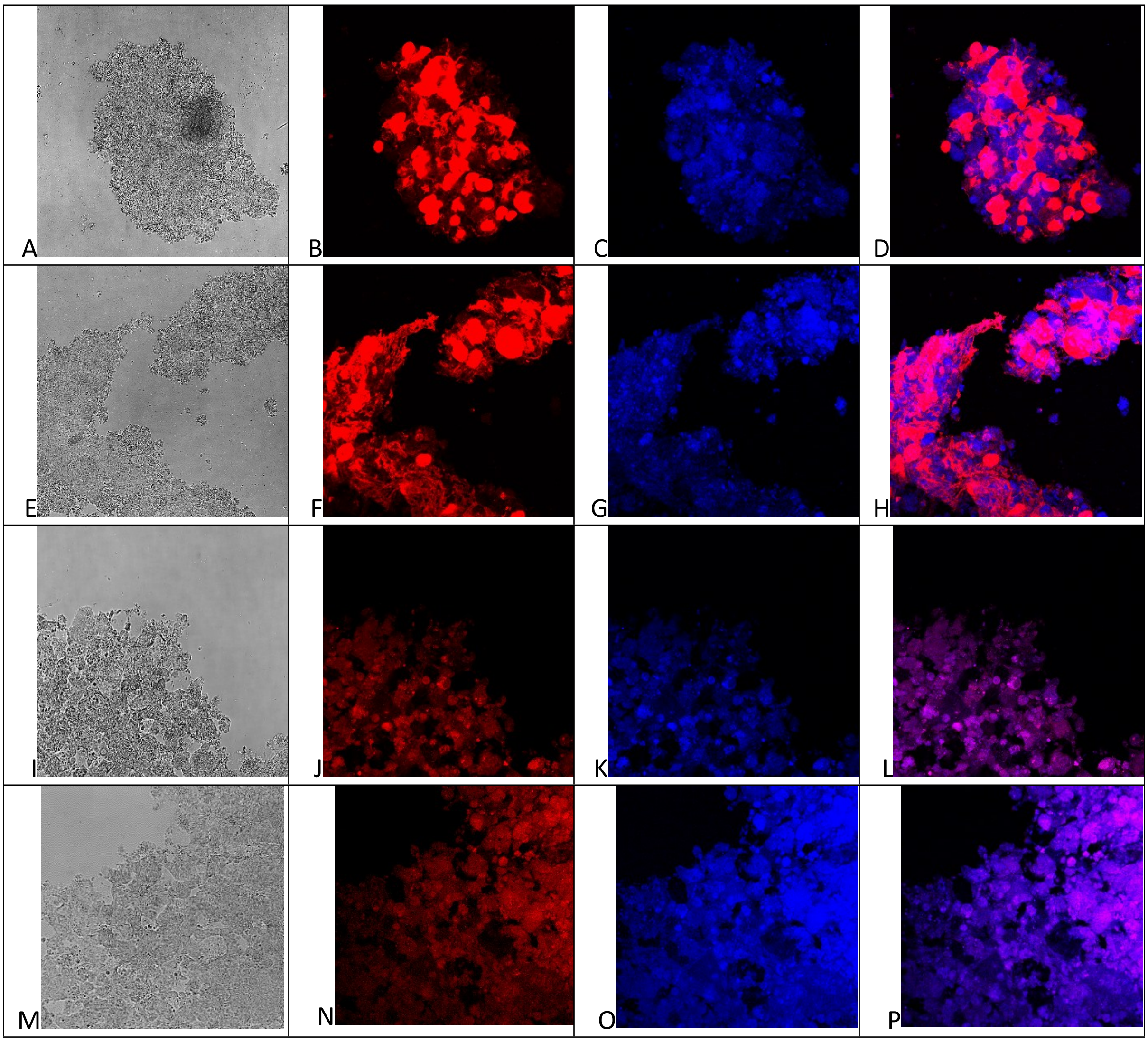

Pieces of matrix with (A-H) and without (I-P) propidium iodide staining. A, E, I, M – bright field images. B, F, J, N – red channel imaging of nuclei with DNA (B, F) or the background autofluorescence (J, N). C, G, K, O – blue autofluorescence. D, H, L, P – merged blue and red images.

References

1 V. W. C. Wong, D. G. Reid, W. Y. Chow, R. Rajan, M. Green, R. A. Brooks and M. J. Duer, *J. Biomolec. NMR*, 2015, **63**, 119.

2 W. Y. Chow, R. Rajan, K. H. Muller, D. G. Reid, J. N. Skepper, W. C. Wong, R. A. Brooks, M. Green, D. Bihan, R. W. Farndale, D. A. Slatter, C. M. Shanahan and M. J. Duer, *Science*, 2014, **344**, 742.

3 C. Sauvee, M. Rosay, G. Casano, F. Aussenac, R. T. Weber, O. Ouari and P. Tordo, *Angewandte Chemie (Int. Ed.)*, 2013, **52**, 10858.

4 M. Geiger, A. Jagtap, M. Kaushik, H. Sun, D. Stoppler, S. Sigurdsson, B. Corzilius and H. Oschkinat, *Chem. Eur. J.*, 2018, **24**, 13485.

5 G. Metz, X. Wu and S. O. Smith, *J. Magn. Reson. Ser. A*, 1994, **110**, 219.

6 K. Takegoshi, S. Nakamura and T. Terao, *Chem. Phys. Lett.*, 2001, **344**, 631.

7 M. Hohwy, C. M. Rienstra, C. P. Jaroniec and R. G. Griffin, *J. Chem. Phys.*, 1999, **110**, 7983.

8 C. P. Jaroniec, C. Filip and R. G. Griffin, *J. Amer. Chem. Soc.*, 2002, **124**, 10728.

9 J. Schaefer, R. A. McKay and E. O. Stejskal, *J. Magn. Reson.*, 1979, **34**, 443.

10 M. Baldus, A. T. Petkova, J. Herzfeld and R. G. Griffin, *Molec. Phys.*, 1998, **95**, 1197.

11 S. Cadars, J. Sein, L. Duma, A. Lesage, T. N. Pham, J. H. Baltisberger, S. P. Brown and L. Emsley, *J. Magn. Reson.,* 2007, **188**, 24.

12 W. Y. Chow, R. Li, I. Goldberga, D. G. Reid, R. Rajan, J. Clark, H. Oschkinat, M. J. Duer, R. Hayward and C. M. Shanahan, *Chem. Comm.*, 2018, **54**, 12570.
